# Growth Performance and Stress Responses of Larval Mississippi Paddlefish *Polyodon spathula* to Hypoxia under Different Diet Treatments

**DOI:** 10.1155/2014/404595

**Published:** 2014-04-14

**Authors:** Ya Zhu, Qiliang Ding, Wen Lei, Chunfang Wang

**Affiliations:** College of Fisheries, Huazhong Agricultural University, Shizishan Road, Hongshan District, Wuhan, Hubei 430070, China

## Abstract

A growth trial was conducted to detect the effects of different diets on the growth performance and hypoxia adaptation capacity of Mississippi Paddlefish (*Polyodon spathula*) larvae. The larvae were fed with live food, formulated diets, and 1/2 live food with 1/2 formulated diets. After a 15-d growth trial, final body weight and total body length were measured, and five larvae from each dietary group were subjected to 1 h of hypoxia treatment. Serum total antioxidant capacity (T-AOC), serum superoxide dismutase (SOD), and liver malondialdehyde (MDA) were measured. Final body weight and weight gain of the fish fed live food were significantly higher than the values for the other two groups. Total body length of the fish fed live food and 1/2 live food with 1/2 formulated diets exhibited no significant difference. After hypoxia treatment, serum T-AOC and SOD activities of the fish fed formulated diets were significantly lower than those of the other two groups. Liver MDA content of the fish fed with live food was significantly higher than that of the other two groups. In conclusion, larval paddlefish fed with an appropriate proportion of live food and formulated diets exhibit improved adaptive capacity to hypoxia.

## 1. Introduction

The paddlefish,* Polyodon spathula*, is a freshwater chondrostean fish that belongs to the suborder Acipenseroidei (order Acipenseriformes) [1]. This North American relict species is indigenous only to the waters of the Mississippi-Missouri River system and neighboring coastal drainages that flow into the Gulf of Mexico [2]. The paddlefish was first introduced to China in 1988. The high prices for paddlefish roe and flesh have resulted in the recent development of paddlefish aquaculture. Paddlefish primarily feed on zooplankton and occasionally consume small insects, insect larvae, and small fish [3]. Moreover, paddlefish are ram suspension filter-feeders [4]. These features make appropriate food resource a bottleneck in the paddlefish aquaculture, especially in the early life stage. Diet items of larval and juvenile paddlefish include zooplankton and all stages of aquatic insects [3]. Larval paddlefish reared under laboratory conditions readily accept natural prey items (live and frozen), as well as commercially prepared powdered diets [5]. However, according to Chinese farmers, larvae with a total body length of less than 110 mm are difficult to wean to formulated diets. Moreover, the survival and growth rates of such fish were significantly lower than those of fish fed with live zooplanktons.

A variety of environmental factors, such as temperature and oxygen availability, can significantly affect fish metabolism, which is extremely important during fish larvae transportation [6]. Two possible responses to ambient oxygen level reportedly exist. In one approach, oxygen regulators adjust their ventilation rates to compensate for changing oxygen levels and to maintain a constant respiration rate and, thus, aerobic metabolism [7]. In the other approach, oxygen conformers enable a decline in respiration rates as environmental oxygen decreases, with concomitant reductions in metabolic rate and aerobic metabolism [7]. Paddlefish are oxygen regulators and maintain constant respiration rates from 150 mm Hg to 90 mm Hg [8]. Tolerance to hypoxia can be regulated by certain diet components, such as n-3 HUFA [9] and vitamin E [10].

Reactive oxygen species (ROS) is as a natural byproduct of normal oxygen metabolism that includes superoxide anion radical, hydrogen peroxide, and highly potent hydroxyl radical [11, 12]. Free radicals, such as superoxide anion radical and hydroxyl radical, with unpaired electrons on an otherwise open shell configuration, such as superoxide anion radical and hydroxyl radical, are usually highly reactive because they are likely to participate in chemical reactions [13]. The cytotoxic effects of ROS include membrane lipid peroxidation, redox balance alteration, enzyme inactivation, and DNA damage [14, 15]. Consequently, aerobic organisms have evolved effective defense systems against oxidative damage [16]. Such defense systems consist of both hydrophilic and lipophilic antioxidant compounds or scavengers and specific antioxidant enzymes, including serum superoxide dismutase (SOD), catalase, and glutathione peroxidase [17].

Oxidative stress is a situation in which steady-state ROS concentration is transiently or chronically enhanced. This condition disturbs cellular metabolism and regulation, aside from damaging cellular constituents [18]. Similar to all aerobic organisms, fish are susceptible to ROS attacks and have developed antioxidant defenses, especially adapted enzymes, such as SOD, catalase, and glutathione peroxidase [19]. Changes in antioxidant enzymes and their protective mechanisms are early indicators of cellular susceptibility to oxidant injury caused by ROS [20].

This study used different diet combination to investigate the possibility of weaning larval paddlefish with body length of approximately 55 mm to commercial formulated diets. We hypothesized that larval paddlefish fed with different diets would show different responses to hypoxia.

## 2. Materials and Methods

### 2.1. Animals, Diets, and 15-Day Growth Trial

Mississippi Paddlefish (*P. spathula*) larvae were acquired from Sturgeon Culture Base of Huazhong Agricultural University, MaCheng city, Hubei province, China. When the larvae were hatched, they were transferred to laboratory in oxygenated plastic bags.* P. spathula* (*n* = 270), with a mean ± SE initial mass of 1.14 ± 0.03 g, were randomly distributed to nine tanks with 30 fish per tank, with no significant differences in mean mass among the groups. The larval paddlefish were maintained indoors in 397 L tanks (diameter: 80 cm, height: 70 cm) with a continuous supply of biofiltered freshwater at the Experimental Aquaculture Center in Huazhong Agricultural University from late April to early May. The larvae were exposed to a natural photoperiod of approximately 12 : 12  L : D. The temperature throughout the experimental period was between 24 and 24.8°C, whereas the water flow velocity was approximately 729 mL/min. Actually, the water flow velocity into each tank cannot be exactly the same every day. But we could make the water flow velocity close among the tanks by manually adjusting the water faucet. Total ammonia-nitrogen [(NH_4_^+^ + NH_3_)-N] was always maintained below 0.5 mg/L, and the pH value was about 8.07. Residual chlorine was determined weekly, and levels were consistently below 0.05 mg/L. Dissolved oxygen during the growth trial was maintained at approximately 8.5 mg/L.

Two fish feed were used in the growth trial. One feed was live zooplanktons, mainly cladocera and rotifera, captured from South Lake every morning. The proportions of cladocera, rotifera, and others in our water samples were about 15%, 80%, and 5% by number during the period of our experiment. The other feed was commercially formulated diets for first-feeding fish larvae. The feed we used was brought from the most famous larvae formulated feeds company in China and it was proved to be a good live food replacement for sturgeon larvae (S1 number microparticles of Shengsuo Commercial Formulated Feed, Shandong, China; diameter: 150 *μ*m to 250 *μ*m). The diet composition provided by the feed company was crude protein = 51.6%, crude lipid = 10.2%, ash = 15.2%, calcium = 1.60%, total phosphorus = 1.55%, and moisture = 11.2%. Three feeding strategies were adopted in the 15-day growth trial. The first treatment group was fed completely with live zooplanktons, the second treatment group was fed completely with formulated diets, and the last treatment group was fed with a combination of nearly half live zooplanktons and half formulated diets by biomass. In order to make half live zooplanktons and half formulated diets, we calculated the numbers of cladocera and rotifer (dominated in water samples by biomass) in 10 L water samples first and then collected the zooplanktons from 10 L water samples by filtering the water samples with 45 *μ*m membrane filter, weighted wet weight and dry weight of the filtered zooplanktons. Using the weight and number of zooplanktons of 10 L water samples, after we checked the numbers of cladocera and rotifer of every day's water samples, we then knew how many zooplanktons we should give to the fish comparable to half amount of formulated diets. Each treatment had three replicates, and the diets were fed to the larvae four times a day to satiation (8:00, 12:00, 15:00, and 18:00). Excess feed and feces were collected every morning before feeding (siphoning), and dead larvae were removed and counted twice a day.

Initial body weight and body length data were obtained at the beginning of the growth trial. After the growth trial, five fish were randomly selected from each tank and transferred to another set of tanks (with similar tank size used in growth trial) for future hypoxia treatment. All of the other remaining fish were starved for 24 h and anesthetized with MS-222 (100 mg L^−1^). The fish were then counted, bulk-weighed, and measured (body length) to determine the growth performance parameters.

### 2.2. Hypoxia Treatment

After a 15-day of feeding, five fish from each diet treatment were subjected to low oxygen treatment. Sodium sulfite anhydrous was added into the three tanks with 100 L of experimental water using a dissolved oxygen meter (HQ40d; HACH, USA). After several hours of monitoring and adjustment, the dissolved oxygen concentration in each tank changed from 8.5 mg/L to 3.33 mg/L–3.46 mg/L. The fish were then placed into the tanks and left to stand for 1 h.

After hypoxia treatment, fish were anesthetized by placing them immediately in a concentration (100 mg L^−1^) of MS-222. The five fish were then weighed and measured (body length). For serum samples preparation, blood was withdrawn from the caudal vein into plastic Eppendorf tubes using sterilized syringes and mixed and kept at 4°C for 1 h and then centrifuged (4°C, 3000 g for 10 min). The supernatants were collected as the serum samples and frozen at −20°C for subsequent analysis. Liver samples were dissected from fish, the blood samples if which were obtained. These fish were frozen immediately in liquid nitrogen and stored at −80°C until use. Measurements of enzymatic activities of total antioxidant capacity (T-AOC), SOD, and malondialdehyde (MDA) contents were determined spectrophotometrically using the corresponding kits (Nanjin Jiancheng Bioengineering Institute, Nanjin, China) and following the manufacturer's protocol.

### 2.3. Statistical Analysis

Data are reported as means ± SE. All data means were compared using Duncan's multiple range test after the homogeneity of variances was tested (Statistical Package Social Science, SPSS, version 16.0). ANOVA was performed for the statistical analyses. *P* < 0.05 was considered statistically significant.

## 3. Results

### 3.1. Growth Performance of Larval Paddlefish

Table 1 presents the growth performance of larval paddlefish fed with live food, formulated diets, and 1/2 live food with 1/2 formulated diets.

The final body weight and weight gain of the fish fed with live food were significantly higher than those of the other two groups, whereas no significant difference existed between fish fed with formulated diets and those fed with 1/2 live food with 1/2 formulated diets. The total body length and mortality of the fish fed with live food and 1/2 live food with 1/2 formulated diets exhibited no significant difference. However, these two groups exhibited significantly higher values than the group fed with formulated diets.

### 3.2. Biochemical Indicators in Serum and Liver

Table 2 presents the serum T-AOC and SOD activities, as well as liver MDA content, of larval paddlefish fed with live food, formulated diets, and 1/2 live food with 1/2 formulated diets.

After hypoxia treatment, serum T-AOC and SOD activities of the fish fed with formulated diets were significantly lower than those of the other two groups, whereas no significant difference was observed between fish fed with live food and those fed with 1/2 live food with 1/2 formulated diets. The liver MDA content of the fish fed live food was significantly higher than that of the other two groups. By contrast, no significant difference was found between fish fed with formulated diets and those fed with 1/2 live food with 1/2 formulated diets.

## 4. Discussion

First-feeding larvae generally depend on live food. However, live food is difficult to sustain and requires considerable space and expense; formulated diets are easier to maintain [21]. Thus, formulated diet substitution for live food is crucial for reducing production costs and sustaining production of high- and constant-quality juveniles.

Survival rates and growth performance in larvae were indicative of suitable rearing conditions [22]. In this study, the larvae fed formulated diets had the highest mortality, in agreement with previous work [23].

The larvae fed with formulated diets exhibited the lowest growth performance, which agrees well with other findings observed in* Acipenser persicus* [24]. The results are mainly attributed to an incomplete primitive larval digestive system [25]. Moreover, formulated diets are commonly composed of denatured insoluble proteins and carbohydrates [26]. In this study, the relative denseness and hardness of formulated diets may adversely affect the digestibility of formulated food compared with live food.

This study detected serum T-AOC, serum SOD, and liver MDA to determine hypoxia adaptation capacity after feeding different diets.

SOD is an antioxidant enzymatic defense system that is an important biochemical parameter for antioxidant effects; SOD converts the superoxide radical to hydrogen peroxide [27]. T-AOC reflects the overall cellular endogenous antioxidative capability for both enzymatic and nonenzymatic antioxidants [19]. The results indicate that paddlefish fed with live food and those fed with 1/2 live food with 1/2 formulated diets responded to environmental changes more quickly than those fed with formulated diets.

Liver MDA content of the fish fed with live food was significantly higher than that of the other two groups, whereas no significant difference existed between fish fed with formulated diets and those fed with 1/2 live food with 1/2 formulated diets. The analogous observation was also supported by previous studies [28]. The lipid peroxidation level of* Solea senegalensis* larvae fed with live food was higher than that of the larvae fed with inert food.

Lipid peroxidation is one of the main processes induced by oxidative stress. MDA formation is a widely used assay for lipid peroxidation, which represents the final product of lipid peroxidation [19]. MDA concentration provides direct evidence of the toxic processes caused by free radicals, and MDA level is considered a suitable indicator of the extent of lipid peroxidation [29]. In this study, lipid peroxidation was elevated in the liver of paddlefish after exposure to hypoxia, as evidenced by increased MDA production. This result suggests the participation of free radical-induced oxidative cell injury in mediating hypoxia. Consequently, lipid peroxidation cannot be prevented despite the induction of T-AOC and SOD activities in the group of fish fed with live food. However, the decrease in the MDA level in the group of fish fed with 1/2 live food with 1/2 formulated diets may be an indicator of an increase in the enzymatic and nonenzymatic antioxidants of defense mechanisms.

Formulated diets, as nonliving material, may induce low-intensity oxidative stress in paddlefish. Preexposure to low-intensity oxidative stress, regardless of how the stress is induced, may enhance tolerance and result in higher oxidative stress intensity. This phenomenon is called preadaptation or cross-adaptation approach [30]. However, this adaptation has associated metabolic costs, which includes diverting energy from normal metabolic functions to the functions that are used to cope with stress [31]. Except for the difference in the trophic structure between formulated diets and live food, metabolic cost is another reason for the lower growth performance of paddlefish fed with formulated diets than those fed with live food.

In farm production, any diet that reduces the reliance on live food production is of technical and economic interest in rearing larval paddlefish. All these factors indicate that feeding the larvae with a combination of live food and formulated diets is optimal. Other studies also reported that a mixture of live food and formulated diets can be used at first feeding in* Acipenser fulvescens* [32] and* Acipenser persicus* [24]. However, future experiments are needed to determine the proportion of live food and formulated diets more precisely to reach the optimal growth performance and hypoxia adaptation capacity.

## 5. Conclusion

In conclusion, larval paddlefish fed with an appropriate proportion of live food and formulated diets exhibited improved hypoxia adaptation capacity without affecting growth performance.

## Figures and Tables

**Table 1:** Growth performance of Mississippi paddlefish (*Polyodon spathula*) under different dietary treatments (mean ± SE).

Treatment	IBW (g)	FBW (g)	BL (cm)	WG (%)^1^	Mortality (%)
Initial			5.54 ± 0.07^a^		
Live food	1.14 ± 0.02	5.67 ± 0.11^a^	11.84 ± 0.50^b^	435.31 ± 11.14^a^	15.56 ± 2.94^a^
1/2 Live food + 1/2 formulated food	1.14 ± 0.03	4.33 ± 0.18^b^	11.48 ± 0.33^b^	303.05 ± 25.06^b^	26.67 ± 3.85^a^
Formulated food	1.15 ± 0.03	3.77 ± 0.35^b^	10.34 ± 0.39^c^	246.14 ± 29.06^b^	64.44 ± 7.29^b^

Note: IBW: initial body weight; FBW: final body weight; BL: body length.

^1^WG (%): weight gain (%) = (final weight − initial weight)/initial weight × 100.

Values with different letters within the same column are significantly different (*P* < 0.05).

**Table 2:** Adaptive responses of Mississippi paddlefish (*Polyodon spathula*) to hypoxia under different dietary treatments (mean ± SE).

Treatment	Serum T-AOC (U/mL serum)	Serum SOD (U/mL serum)	Liver MDA (nmol/mgprot)
Live food	19.12 ± 4.97^a^	71.83 ± 7.91^a^	7.61 ± 1.60^a^
1/2 Live food + 1/2 formulated food	12.13 ± 1.93^a^	63.84 ± 5.37^a^	2.93 ± 0.65^b^
Formulated food	6.99 ± 2.08^b^	49.20 ± 3.59^b^	2.98 ± 0.41^b^

Note: T-AOC: total antioxidant capacity; SOD: superoxide dismutase; MDA: malondialdehyde.

Values with different letters within the same column are significantly different (*P* < 0.05).
